# Potentially Toxic Metal Levels in Breast Milk and Their Determinants Among Lactating Mothers of the DSAN-12 M Birth Cohort in the Reconcavo Baiano, Brazil

**DOI:** 10.1007/s12011-026-05316-x

**Published:** 2026-08-27

**Authors:** Erival Amorim Gomes-Junior, Victor Otero Martinez, Homègnon Antonin Ferréol Bah, Nathália Ribeiro dos Santos, Daisy Oliveira Costa, João Vitor Araújo Santana, Vinicius Silva Pitanga de Jesus, Sarah Adriana Rocha Soares, José Antonio Menezes-Filho

**Affiliations:** 1 Graduate Program in Food Science, College of Pharmacy, Federal University of Bahia, Bahia, Brazil; 2 Graduate Program in Pharmacy, College of Pharmacy, Federal University of Bahia, Bahia, Brazil; 3 Graduate Program in Public Health, Federal University of Bahia, Salvador, Bahia Brazil; 4 Laboratory of Toxicology, College of Pharmacy, Federal University of Bahia, Bahia, Brazil; 5 Laboratory of Excellence in Geochemistry: Oil, Energy and Environment, Federal University of Bahia, Bahia, Brazil

**Keywords:** Biomonitoring, Maternal-child health, Breast milk, Toxic metals, breastfeeding, environmental exposure

## Abstract

Potentially toxic metals (PTMs) can be transferred from mothers to infants through breast milk (BM), representing an important route of early-life exposure. Although PTM concentrations in breast milk have been extensively investigated, relatively few studies have evaluated the environmental determinants associated with these concentrations, particularly in prospective birth cohorts from socially vulnerable populations. This study aimed to determine PTM concentrations in BM and identify environmental, sociodemographic, and dietary determinants associated with these concentrations among lactating women from the prospective Socioenvironmental Determinants of Child Neurodevelopment at 12 Months (DSAN-12 M) birth cohort in northeastern Brazil. BM samples were collected from 48 exclusively breastfeeding women and analyzed for arsenic (As), cadmium (Cd), mercury (Hg), manganese (Mn), and lead (Pb) using inductively coupled plasma tandem mass spectrometry (ICP-MS/MS). Independent determinants of BM metal concentrations were investigated using multivariable linear regression models. Median concentrations (µg/L) were 1.22 for As, 0.42 for Cd, 3.58 for Hg, 5.56 for Mn, and 14.34 for Pb. Over 65% exceeded internationally proposed reference values for As, Hg, Mn, and Pb, whereas 25% exceeded the reference value for Cd. Household water supply was independently associated with Pb and Mn concentrations, household waste burning with Mn concentrations, and municipality of residence with Hg concentrations after multivariable adjustment. These findings indicate widespread co-exposure to multiple PTMs among lactating women in this socially vulnerable population and suggest that structural environmental conditions are important determinants of maternal exposure. Strengthening environmental infrastructure, human biomonitoring, and environmental health policies may reduce maternal and infant exposure to PTMs.

## Introduction

Breast milk (BM) is recognized as the optimal source of nutrition for infants, providing not only essential macronutrients and micronutrients but also a complex array of bioactive compounds, immune factors, hormones, and growth modulators that support gastrointestinal maturation, immune development, and optimal growth during early life [1–3]. Beyond its nutritional role, BM has increasingly been recognized as a valuable biomonitoring matrix, reflecting maternal exposure to environmental contaminants, including potentially toxic metals (PTMs), that may be transferred to the infant during lactation [4, 5].

The World Health Organization recommends exclusive breastfeeding during the first six months of life, followed by continued breastfeeding alongside complementary foods up to two years of age or beyond [6]. During this period, breast milk constitutes the primary source of nutrient intake and, consequently, an important route of infant exposure to both essential trace elements and environmental contaminants [6, 7].

Although breast milk remains the optimal source of infant nutrition, it has also been increasingly recognized as an important biomonitoring matrix for assessing maternal and early-life exposure to environmental contaminants [4, 5]. Recent studies have consistently demonstrated that potentially toxic metals (PTMs), including arsenic (As), cadmium (Cd), mercury (Hg), lead (Pb), and manganese (Mn), can be detected in human milk, reflecting both maternal body burden and ongoing environmental exposure [7, 8]. The transfer of these elements during lactation has emerged as an important public health concern because breastfed infants may be exposed during a critical window of rapid growth and neurodevelopment [5, 9]. Furthermore, marked geographic variability in PTM concentrations has been reported worldwide, suggesting that environmental contamination, dietary habits, industrial activities, and socioeconomic conditions substantially influence metal concentrations in breast milk [8, 10, 11].

Among the trace elements detected in human milk, manganese (Mn) is an essential micronutrient required for normal growth, bone formation, and neurodevelopment. In contrast, arsenic (As), cadmium (Cd), mercury (Hg), and lead (Pb) have no known physiological function and are recognized as priority environmental contaminants due to their persistence, potential bioaccumulation, and adverse effects on human health [10, 12–17]. Human milk may therefore contain both essential and toxic elements, reflecting maternal nutritional status and environmental exposure. Increasing evidence indicates that PTMs can be transferred from the mother to the infant during lactation, making breast milk an important route of postnatal exposure and a valuable indicator of maternal body burden [4, 7, 8]. This exposure is particularly relevant during infancy, a critical developmental period characterized by rapid brain growth, organ maturation, and metabolic programming, in which immature detoxification systems and physiological barriers may increase susceptibility to environmental contaminants. Although Mn is physiologically essential, excessive exposure may impair neurological function. In contrast, exposure to As, Cd, Hg, and Pb has been associated with adverse outcomes including impaired neurodevelopment, reduced cognitive performance, behavioral alterations, oxidative stress, and endocrine dysfunction [4, 14, 18–22]. Consequently, understanding the occurrence and determinants of PTMs in breast milk has become an important public health priority, particularly in populations exposed to multiple environmental sources of contamination, while preserving the well-established benefits of breastfeeding [5, 11].

Numerous studies conducted across Europe, Asia, Latin America, and Africa have consistently demonstrated that concentrations of potentially toxic metals in human milk vary substantially among populations, reflecting differences in environmental contamination, dietary habits, industrial activities, socioeconomic conditions, and public health infrastructure [4, 8, 10, 11]. Elevated concentrations of Pb, Cd, Hg, and As have been reported in countries such as China, Iran, and several regions of Brazil, reinforcing concerns regarding postnatal exposure during breastfeeding [4, 23–25]. Despite the growing body of evidence describing PTM concentrations in breast milk, comparatively fewer studies have investigated the environmental, socioeconomic, and dietary determinants influencing maternal transfer of these contaminants, particularly using prospective birth cohort designs in socially vulnerable populations. Recently, our research group reported elevated exposure to Cd, Mn, and Pb among pregnant women enrolled in the DSAN-12 M birth cohort, highlighting the need to investigate whether maternal exposure is reflected in breast milk and to identify the factors associated with this transfer [22].

The Recôncavo Baiano region, located in the state of Bahia, northeastern Brazil, is characterized by environmental diversity and a history of contamination [22, 26, 27]. Despite undergoing industrial and agricultural development, many communities in the region remain socially vulnerable [22]. These vulnerabilities, together with environmental stressors such as proximity to factories, mining sites, highways, and agricultural activities, may increase the risk of human exposure to toxic substances [28]. Therefore, this region provides an important setting for investigating maternal exposure to PTMs and their transfer to breast milk. However, evidence regarding the determinants of PTM concentrations in human milk from this region remains scarce.

This study aimed to investigate the concentrations of potentially toxic metals (PTMs) in breast milk from lactating women enrolled in the DSAN-12 M birth cohort and to identify the environmental, sociodemographic, and dietary determinants associated with these concentrations. By addressing an important gap in the literature on the determinants of PTM transfer to human milk in a socially vulnerable population, this study seeks to improve understanding of environmental exposure pathways during lactation and to provide evidence to support targeted public health strategies to reduce maternal and infant exposure to toxic metals.

## Methodology

### Study Design and Population

**Fig. 1:**
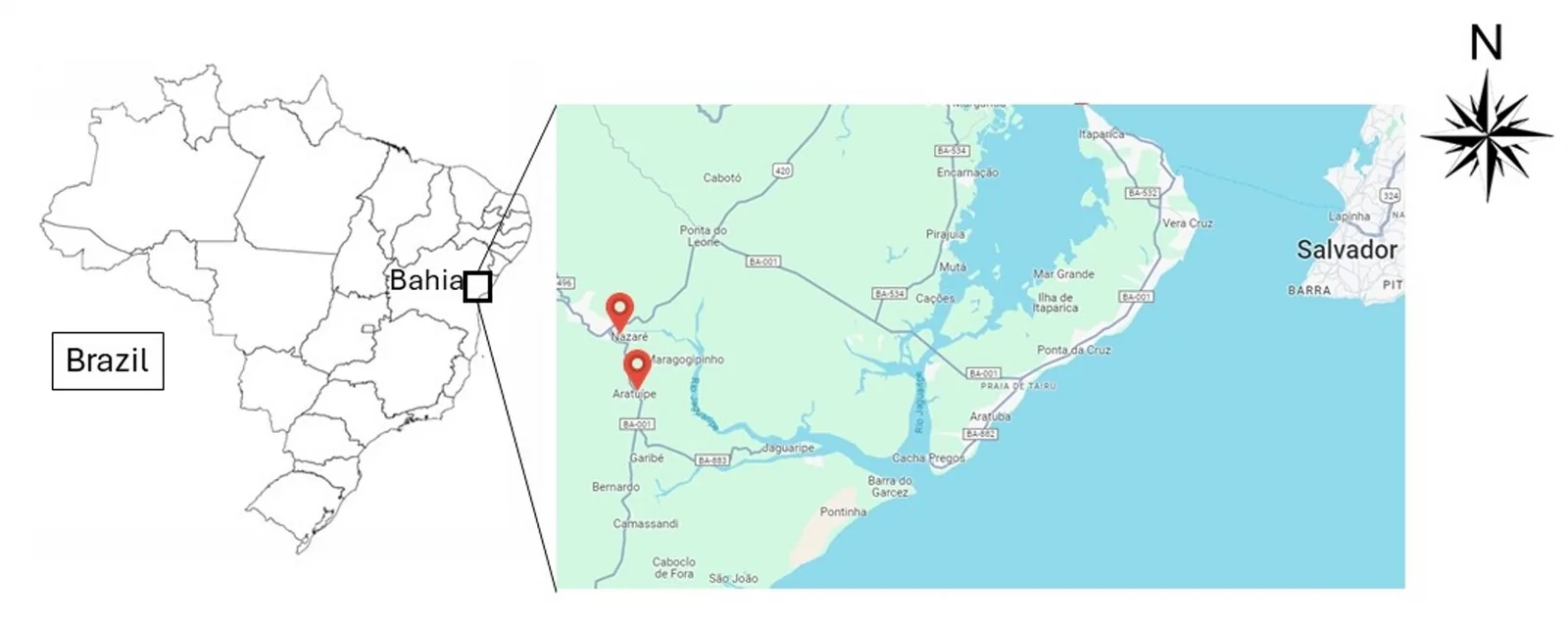
Schematic map showing the study area (red markers)

This prospective cohort study is part of the Socioenvironmental Determinants of Child Neurodevelopment at 12 Months (DSAN-12 M) birth cohort study conducted in two municipalities (Aratuípe and Nazaré) in the Recôncavo Baiano region, Brazil (Fig. 1). These municipalities are characterized by social vulnerability and environmental exposure to potentially toxic metals [21, 22]. In addition to the social vulnerability faced by the population, the presence of factories and quarries, proximity to highways, and activities such as lead-glazing ceramics and agriculture may contribute to environmental and human exposure to PTMs.

The DSAN-12 M birth cohort consists of pregnant women and their newborn infants, who were recruited and followed up to investigate the effects of environmental exposures to toxic compounds on pregnant women and their children’s neurodevelopment up to 12 months. A total of 102 pregnant women were enrolled in the DSAN-12 M birth cohort. Among them, 48 lactating women provided breast milk samples and were included in the present analysis.

Participants were recruited during second-trimester prenatal consultations and followed from pregnancy through the postpartum period. Written informed consent was obtained from all participants prior to study enrollment. Following enrollment, trained members of the research team conducted home visits to collect information on sociodemographic characteristics, medical history, dietary habits, and biological samples (blood, urine, occipital hair, and breast milk).

Breast milk samples were collected only from participants who were exclusively breastfeeding at the time of collection. Women who had discontinued exclusive breastfeeding were not eligible for breast milk sampling. This approach aimed to ensure that breast milk samples reflected infant exposure during the period of exclusive breastfeeding, which is considered critical for early-life exposure assessment.

## Data Collection

Semi-structured questionnaires were administered by trained researchers to the study participants until the end of the second trimester of pregnancy. The sociodemographic and environmental questionnaire was completed by 102 participants, while the semi-quantitative food frequency questionnaire (FFQ) was completed by 64 participants. Data on general daily habits, education level, occupational history, medical history, smoking habits in the residence, and proximity to probable sources of exposure to potentially toxic metals were then collected. Diet was assessed by a semi-quantitative food frequency questionnaire (FFQ) applied by a trained dietitian during the second trimester.

## Breast Milk Sampling

A total of 48 breast milk samples were obtained from the lactating women included in the study. Before breast milk sample collection, lactating women were instructed and supervised by trained researchers to perform standardized breast hygiene procedures. These procedures included tying back hair, washing hands with neutral soap, followed by sanitization with 70% ethyl alcohol, and cleaning the nipples and areolas using water or moistened wipes. Breast milk was collected by manual expression by the lactating women with the assistance of researchers. Participants were asked to provide up to 40 mL of breast milk; however, the volume obtained varied among participants, particularly during the early months of lactation, when some women were unable to provide the requested volume. Samples were collected during the period of exclusive breastfeeding, up to the sixth month postpartum or until exclusive breastfeeding was discontinued. Samples were collected directly into 80 mL polyethylene flasks, which were previously decontaminated with 10% (v/v) HNO_3_, stored in ice boxes, transferred to the Laboratory of Toxicology (LABTOX) at the Federal University of Bahia, and kept in an ultra-freezer at -70 °C until further analytical processing.

## Equipment and Reagents

Samples were weighed in an analytical balance (CP2245 Sartorius^®^, Germany). The freeze-drying process was carried out by lyophilization (Freeze dryer Liotop L101, São Paulo, Brazil). The samples were digested in a microwave-assisted digestion oven (MARS-6, CEM^®^, USA). Digested samples were transferred to 15-mL polypropylene centrifuge tubes (Corning^®^ CentriStar™, St. Louis, MO, USA). For sample digestion, we used ultrapure nitric acid (69% v/v) (JTBaker^®^ Deventer, Netherlands), Hydrogen Peroxide (30%) (Merck^®^, Darmstadt, Germany), and ultrapure water obtained by the Milli-Q system of treatment (Millipore^®^, Bedford, USA). Metal analysis of the breast milk samples was performed by inductively coupled plasma mass spectrometry (ICP-MS/MS) (Agilent^®^ 8800 Triple Quadrupole ICP-MS/MS, Santa Clara, CA, USA). Human milk certified reference material (ERM^®^-BD150) was acquired from European Commission’s Joint Research Centre (Geel, Belgium).

## Analytical Procedure

Human milk samples were previously aliquoted, and each aliquot was weighed prior to lyophilization. Aliquots averaging approximately 19 g per sample (wet weight) were transferred to Petri dishes and freeze-dried. For samples with insufficient volume, the entire sample was lyophilized to ensure maximum recovery. After 16 h of lyophilization, the samples were weighed again to determine their moisture content, then digested in a microwave-assisted digestion system.

Although samples were lyophilized for the analytical procedure, moisture content was determined for each sample, and the results were recalculated and expressed on a wet-weight basis (µg/L), allowing direct comparison with international reference values and other studies.

For mineralization, 0.3 g of each lyophilized sample was directly weighed into the Teflon flask vessels of the microwave digester oven. Then, 4 mL of ultrapure nitric acid and 1 mL of hydrogen peroxide were added to the Teflon flask vessels. Microwave-assisted digestion was performed under the following conditions: power, 1030–1800 W; temperature, 210 °C; and digestion time, 40 min. Certified reference material (CRM) and reagent blanks were simultaneously processed with the human milk samples. After cooling to room temperature, the digested samples were transferred volumetrically to a 25 mL flask.

To ensure the accuracy, precision, and reliability of the analysis, rigorous quality control and quality assurance measures were performed. The limits of detection (LODs) were determined by analyzing 10 independent blank solutions using the standard deviation (σ) of multiple blank sample measurements and the slope of the calibration curve, applying the formula LOD = 3σ/slope. The limits of quantification (LOQs) were calculated using the same blank measurements and calibration curves, according to the formula: LOQ = 10 × σ/slope [29, 30]. The accuracy and precision of the method were assessed through a recovery study. The accuracy was assessed using the certified ERM^®^-BD150 Skimmed Milk Powder reference material from the European Commission’s Joint Research Center in Belgium. The acceptance criteria range from 80% to 120% of the certified value, with a maximum relative standard deviation of less than 30% [29, 30].

Table 1 describes the main ICP-MS/MS parameters obtained during the optimization of the methodology used to measure PTMs in breast milk samples, and Table 2 summarizes the method quality assurance results.

**Table 1:** ICP-MS/MS operation conditions

Parameters	ICP-MS/MS operation conditions
Equipment	Agilent 8800 Triple Quadrupole ICP-MS/MS
RF power (W)	1550
Nebulizer	*Micromist*
Auxiliary gas flow rate (L/min)	0.90
Coolant gas flow rate (L/min)	15
Nebulizer gas flow (L/min)	1
Integration time (s)	0.1
RF *matching* (V)	1.8
Collision cell	He 5.0 mL min^− 1^
Acquisition mode	*Spectrum*
Number of replicates	3
*Sweeps*	100
Internal standard	^72^Ge, ^115^In, ^205^Tl
Calibration standards (µg L^− 1^)	0,1 a 100
Isotopes	^55^Mn, ^75^As, ^111^Cd, ^206^Pb, ^201^Hg

**Table 2:** Parameters of the quality assurance of the analytical method

				CRM levels (ERM^®^- BD150)**		
Metal	LOD (µg/L)		LOQ (µg/L)	Certified (mg.kg^− 1^)	Measured (mg.kg^− 1^)	Recovery (%)
As	0.060		0.200	-*	-*	-*
Cd	0.012		0.040	0.0114	0.0113	99.1
Hg	0.053		0.177	0.060	0.061	101.6
Mn	0.024		0.080	0.289	0.264	91.35
Pb	0.015		0.050	0.019	0.018	94.73

*The CRM was not certified to As levels.

** LOD and LOQ reported in µg/L for the analytical solution after sample preparation. Certified/measured values for ERM®-BD150 are provided in mg/kg as per certificate and are not directly comparable without conversion using sample mass and final volume

### Data Analysis

The distributions of continuous variables were evaluated using the Kolmogorov-Smirnov and Shapiro-Wilk test. Continuous variables were described as mean (± standard deviation), and median (interquartile range) according to their distribution.

Because breast milk metal concentrations presented a non-normal distribution, they were log-transformed and analyzed as continuous variables using multivariate linear regression models. Variables were selected based on biological plausibility and epidemiological relevance. Potential confounders were additionally assessed during model building by examining whether their inclusion materially changed the regression coefficients or the statistical significance of the variables retained in the model. Maternal educational level was evaluated as a potential confounder; however, its inclusion did not change the estimated coefficients or the significance of the associations with Pb and Mn, and it was therefore not retained in the final models. Regression coefficients (β), 95% confidence intervals (95% CI), and adjusted R² values were reported.

As a complementary analysis, breast milk metal levels were dichotomized for two purposes. First, to define the proportion (95%; Confidence Interval) of participants with BM levels above the reference values suggested in the literature. Then, the medians were used as cut-off points to dichotomize the metal levels in BM, which were used as dependent variables to define the determinants associated with values above the median. Cut-off points of BM metal levels were 0.6 µg/L (As); 1 µg/L (Cd); 1.7 µg/L (Hg); 4 µg/L (Mn), and 5 µg/L (Pb) [31].

The chi-square test (χ²) was used to compare the frequencies of participants exceeding the reference values according to dietary and sociodemographic characteristics. Univariate logistic regression was performed to estimate the odds ratios (ORs) and 95% confidence intervals (95% CIs) for factors associated with elevated BM metal levels. Spearman’s correlation analysis was employed to assess potential correlations among BM metal levels.

Statistical analyses were performed using IBM SPSS Statistics for Windows, version 26, and a p-value < 0.05 was considered statistically significant.

## Results

### Sociodemographic and Nutritional Characteristics of the Study Population

Table 3 summarizes the sociodemographic characteristics of the study population, comprising 102 pregnant women enrolled in the DSAN-12 M birth cohort. Of these, 64.7% lived in the municipality of Nazaré and 35.3% in Aratuipe. A statistically significant difference was observed in the distribution by municipality between participants who provided breast milk samples and those who did not (*p* = 0.03), with a higher proportion of BM samples collected from participants living in Aratuipe (45.8%) than those living in Nazaré (54.2%). Regarding ethnicity, 94.1% of the study population self-identified as Black or mixed race, whereas 5.9% identified as White or other ethnic groups; no significant difference between groups (*p* = 0.21). Most participants were between 20 and 34.9 years (75.5%), followed by those under 20 years (13.7%) and women over 35 years (10.8%), with statistically significant difference among them.

Regarding obstetric history, 61.8% of the women were multiparous. A monthly family income of less than or equal to one minimum salary was reported by 61.1% of participants, reflecting the socioeconomic vulnerability of the study population. Concerning household waste disposal, 70.6% of participants reported having access to public waste collection. The remaining (29.4%) reported burning their household waste (*p* = 0.073). Household exposure to secondhand tobacco smoke was reported by 19.3% of participants, and a public water system supplied the majority (84.6%) of households.

Only the municipality of residence showed a significant difference between the groups that provided BM samples and those that did not. The absence of significant differences in the remaining sociodemographic and environmental characteristics suggests that the study population was generally homogeneous, particularly with respect to socioeconomic and environmental factors.

**Table 3:** Sociodemographic characteristics of the study population

Variables (characteristics)		*N* (%)			*p*-value
		Total	Non-sampled	sampled	
Municipalities (*n* = 102)	Aratuípe	36 (35.3)	14 (25.9)	22 (45.8)	0.03*
	Nazaré	66 (64.7)	40 (74.1)	26 (54.2)	
Ethnicity (*n* = 102)	White/Other	6 (5.9)	5 (9.3)	1 (2.1)	0.21
	Black/Brown	96 (94.1)	49 (90.7)	47 (97.9)	
Age group (years) (*n* = 102)	< 20	14 (13.7)	7 (13.0)	7 (14.6)	0.747
	20–34.9	77 (75.5)	40 (74.1)	37 (77.1)	
	> 35	11 (10.8)	7 (13.0)	4 (8.3)	
Birth History (*n* = 89)	Primiparous	35 (38.2)	18 (40.9)	16 (35.6)	0.603
	Multiparous	55 (61.8)	26 (59.1)	29 (64.4)	
Family Income (*n* = 90)	≤ 1 salary	55 (61.1)	26 (57.8)	29 (64.4)	0.517
	> 1 salary	35 (38.9)	19 (42.2)	16 (35.6)	
Waste treatment (*n* = 102)	Collected by public services	72 (70.6)	34 (63.0)	38 (79.2)	0.073
	Waste burn	30 (29.4)	20 (37.0)	10 (20.8)	
Passive smoker (*n* = 88)	No	71 (80.7)	32 (74.4)	39 (86.7)	0.146
	Yes	17 (19.3)	11 (25.6)	6 (13.3)	
House Water Supply (*n* = 84)	Artesian wells and rivers	14 (15.4)	8 (20.5)	6 (13.3)	0.379
	Public water system	70 (84.6)	31 (79.5)	39 (86.6)	

Data in Table 4 summarizes the nutritional and dietary characteristics of the study population, based on the availability of BM samples. Regarding pre-pregnancy BMI, a slight majority of participants were overweight (51.2%), followed by normal weight (43.9%) and underweight (4.9%), with no significant differences between groups. All participants reported daily vegetable consumption. Daily fruit consumption was reported by 78.1% of participants, while weekly consumption was reported by 21.9%, with no significant difference between groups (*p* = 0.752). For the remaining food groups, such as regional dishes (*p* = 0.749), milk and dairy products (*p* = 0.679), bakery products (*p* = 0.502), coffee (*p* = 0.997), meat and eggs (*p* = 0.958), seafood (*p* = 0.247), grains (*p* = 0.136), pasta (*p* = 0.942), manioc flour (*p* = 0.633), and farinaceous products (*p* = 0.678), no significant differences were observed between groups. Overall, the dietary profile was similar between women who provided BM samples and those who did not, indicating that the analyzed subgroup was representative of the original cohort with respect to nutritional and dietary characteristics.

**Table 4:** Nutritional and dietetic characteristics of the study population, according to breast milk sample availability

Variables (characteristics)		*N* (%)			*p*-value
		Total	Non-sampled	sampled	
BMI (Kg/m²) (*n* = 82)	< 18.5	4 (4.9)	17 (44.7)	19 (43.2)	0.974
	18.5–24.9	36 (43.9)	2 (5.3)	2 (4.5)	
	> 25	42 (51.2)	19 (50.0)	23 (52.3)	
*Dietary intake*					
Fruits	Never				0.725
	< 1x/week				
	Weekly	14 (21.9)	6(20)	8(23.5)	
	Daily	50 (78.1)	24(80)	26(76.5)	
Vegetables	Never				-*
	< 1x/week				
	Weekly				
	Daily	64 (100)	30(100)	34(100)	
Oilseed	Never	9 (14.1)	1(3.3)	8(23.5)	0.035
	< 1x/week	30 (46.9)	17(56.7)	16(47.1)	
	Weekly	23 (35.9)	10(33.3)	9(26.5)	
	Daily	5 (7.8)	2(6.7)	1(2.9)	
Desserts	Never	6 (9.4)	5(16.7)	1(2.9)	0.008
	< 1x/week	4 (6.3)	15(50.0)	14(44.1)	
	Weekly	30 (46.9)	9(30.0)	14(41.2)	
	Daily	5 (7.8)	1(3.3)	4(11.8)	
Regional dishes	Never	10 (15.6)	4(13.3)	6(17.6)	0.749
	< 1x/week	39 (60.9)	20(66.7)	19(55.9)	
	Weekly	13 (20.3)	6(20.0)	7(20.6)	
	Daily	2 (3.1)		2(5.9)	
Milk and dairy products	Never	4(6.3)	2(6.7)	2(5.9)	0.679
	< 1x/week	1(1.6)	1(3.3)		
	Weekly	13(20.3)	7(23.3)	6(17.6)	
	Daily	46(71.9)	20(66.7)	26(76.5)	
Bakery products	Never	2(3.1)	2(6.7)		0.502
	< 1x/week	1(1.6)		1(2.9)	
	Weekly	23(35.9)	12(40)	11(32.4)	
	Daily	38(59.4)	16(53.3)	22(64.7)	
Coffee	Never	26(40.6)	12(40.0)	14(41.2)	0.997
	< 1x/week	1(1.6)	1(3.3)		
	Weekly	7(10.9)	3(10.0)	4(11.8)	
	Daily	30(46.9)	14(46.7)	16(47.1)	
Eggs e Meat	Never	1(1.6)	1(3.3)		0.958
	< 1x/week				
	Weekly	16(25)	7(23.3)	9(26.5)	
	Daily	47(73.4)	22(73.3)	25(73.5)	
Seafood	Never	3(4.7)	3(10.0)		0.247
	< 1x/week	33(51.6)	14(46.7)	19(55.9)	
	Weekly	26(40.6)	12(40.0)	14(41.2)	
	Daily	2(3.1)	1(3.3)	1(2.9)	
Grains	Never	1(1.6)	1(3.3)		0.136
	< 1x/week				
	Weekly	14(21.9)	4(13.3)	10(29.4)	
	Daily	49(76.6)	25(83.3)	24(70.6)	
Pasta	Never	3(4.7)	2(6.7)	1(2.9)	0.942
	< 1x/week	14(21.9)	7(23.3)	7(20.6)	
	Weekly	44(68.8)	20(66.7)	24(70.6)	
	Daily	3(4.7)	1(3.3)	2(5.9)	
Manioc flour	Never	15(23.4)	6(20.0)	9(26.5)	0.633
	< 1x/week	7(10.9)	2(6.7)	5(14.7)	
	Weekly	16(25)	8(26.7)	8(23.5)	
	Daily	26(40.6)	14(46.7)	12(35.3)	
Farinaceous	Never	4(6.3)	2(6.7)	2(5.9)	0.678
	< 1x/week	2(3.1)	1(3.3)	1(2.9)	
	Weekly	28(43.8)	11(36.7)	17(50)	
	Daily	30(46.9)	16(53.3)	14(41.2)	

### Descriptive Levels of As, Cd, Hg, Mn and Pb in Breast Milk (BM)

The levels of potentially toxic metals in breast milk samples are summarized in Table 5. BM metal concentrations are presented on wet weight (µg/L), after correction for the use of the moisture content determined during the lyophilization process. All PTMs showed a nonparametric distribution according to the Kolmogorov-Smirnov test and are therefore reported as medians and interquartile ranges (IQR). Arsenic presented a median of 1.22 µg/L (Q1-Q3: 0.81–1.74), reflecting moderate exposure in most participants; however, 89.6% of the samples presented concentrations above the reference limit established by international health agencies, indicating a high prevalence of elevated exposure. For Cd, the median was 0.42 µg/L (Q1-Q3: 0.041–1.12), with a wide dispersion of values, indicating that, although most samples had lower levels, some BM presented high concentrations; notably, 25% of the participants exhibited Cd levels above the recommended limit. Regarding Hg, the median was 3.58 µg/L (Q1-Q3: 1.34–5.62), a value higher than the maximum tolerable limit established by international agencies, suggesting significant exposure among the lactating participants, with 75% of the samples exceeding the recommended threshold. Manganese had a median of 5.56 µg/L (Q1-Q3: 3.01–11.03), also above the reference limit of 4.0 µg/L, with 65,3% of the samples above this threshold. This may indicate excessive exposure of a relevant proportion of women. Lead (Pb) had the highest median among the elements analyzed, with 14.34 µg/L (Q1-Q3: 6.69–21.02), a value almost three times higher than the limit of 5 µg/L, indicating widespread exposure and potential risk for infants, as 83.3% of the breast milk samples exceeded the international safety limit.

**Table 5:** Descriptive levels of, As, Cd, Hg, Mn, and Pb in breast milk samples and percentage of those above the reference values

PTM			Median (µg/L)	Q1-Q3	Reference value (µg/L)	*N*	%
**As**			1.22	0.81–1.74	0.6	43	89.6
**Cd**			0.42	0.041–1.12	1.0	12	25
**Hg**			3.58	1.34–5.62	1.7	36	75
**Mn**			5.56	3.01–11.03	4.0	32	65.3
**Pb**			14.34	6.69–21.02	5.0	40	83.3

*IIQ (Q1 - Q3)

A significant proportion of the breast milk samples presented metal levels above the established reference values. Metal levels in the BM samples were higher than the reference values in the following order: As > Pb> Hg > Mn> Cd, varying from 89.6% to 25%. It is noteworthy that 89.6% (95% CI: 77.8–95.5) of participants had arsenic levels above the 0.6 µg/L limit, while lead levels were elevated in 83.3% (95% CI: 70.4–91.3) of samples. Mercury was also detected above the limit of 1.7 µg/L in 75% (95% CI: 61.2–85.1) of the participants. Manganese was elevated in 65.3% (95% CI: 51.3–77.1), and cadmium, although to a lesser extent, presented levels above the limit in 25% (95% CI: 14.9–38.8) of the samples. These data indicate that a large proportion of samples exceeded the proposed reference values with potential toxicity, which may pose a risk to infant health, given that breast milk is the main source of nutrition in the first months of life.

### Correlations Among Potentially Toxic Metals in Breast Milk

Spearman’s correlation coefficients are summarized in Table 6. Significant positive correlations were observed among PTMs in breast milk. Manganese (Mn) levels showed a moderate positive correlation with both arsenic (As) (rho = 0.335, *p* = 0.020) and lead (Pb) (rho = 0.428, *p* = 0.002). In addition, a significant positive correlation was found between cadmium (Cd) and lead (Pb) levels (rho = 0.364, *p* = 0.011), reinforcing the hypothesis of co-exposure to toxic metals in the studied population. These associations may reflect common sources of contamination, such as environmental pollutants, particularly in socially vulnerable regions. The observation of such correlations underscores the need for integrated monitoring of multiple PTM exposures in breast milk better to understand exposure patterns and potential health risks to infants.

**Table 6:** Spearman correlation coefficients among potentially toxic metals in breast milk

		Mn	As	Cd	Hg	Pb
**Mn**	Rho	1.000				
	*P*					
**As**	Rho	**0.335**	1.000			
	*P*	**0.020**				
**Cd**	Rho	0.268	0.278	1.000		
	*P*	0.065	0.055			
**Hg**	Rho	0.013	-0.195	-0.260	1.000	
	*P*	0.931	0.183	0.074		
**Pb**	Rho	**0.428**	0.123	**0.364**	-0.047	1.000
	*P*	**0.002**	0.407	**0.011**	0.752	

### Sociodemographic and Dietary Determinants of Pb, Mn, and Hg Levels in Breast Milk

Multivariate linear regression models using log-transformed metal concentrations were fitted to investigate environmental, sociodemographic, and dietary determinants associated with Pb, Mn, and Hg levels in breast milk (Table 7). In addition, logistic regression analyses using international reference values as cut-off points were conducted as complementary analyses to investigate factors associated with BM metal concentrations exceeding the international recommended limits (Table 8).

**Table 7:** Multivariate linear regression models for determinants of log-transformed Pb, Mn, and Hg concentrations in breast milk

**log-transformed Pb**			
**Variable**	**Non-standardized β coefficients**	**CI-95%**	***p*** **-value**
Ethnicity	0.130	-0.218–0.479	0.455
Age (years)	0.006	-0.049–0.062	0.824
Birth History	-0.163	-0.924–0.598	0.668
Family Income	0.190	-0.236–0.615	0.373
Waste treatment	0.852	-1.759–0.055	0.065
Passive smoker	0.460	-0.607–1.526	0.390
House Water Supply	1.048	0.105–1.992	0.030*
**log-transformed Mn**			
**Variable**	**Non-standardized β coefficients**	**CI-95%**	***p*** **-value**
Ethnicity	0.071	-0.210–0.352	0.613
Age (years)	-0.009	-0.054–0.036	0.687
Birth History	-0.288	-0.886–0.310	0.336
Family Income	0.247	-0.091–0.585	0.148
Waste treatment	1.338	-1.979 – -0.697	0.001*
Passive smoker	0.081	-0.694–0.856	0.834
House Water Supply	0.943	0.194–1.691	0.015*
Milk and dairy products intake	0.004	-0.018–0.026	0.701
**log-transformed Hg**			
**Variable**	**Non-standardized β coefficients**	**CI-95%**	***p*** **-value**
Ethnicity	-0.276	-0.626–0.074	0.120
Age (years)	-0.032	-0.089–0.024	0.255
Birth History	0.597	-0.161–1.354	0.120
Family Income	-0.319	-0.749–0.111	0.142
Waste treatment	0.254	-0.714–1.221	0.600
Passive smoker	-0.557	-1.651–0.537	0.311
House Water Supply	-0.246	-1.267–0.776	0.631
Municipality	-0.824	-1.491 – -0.157	0.017*

BM: breast milk; CI: confidence interval; β: non-standardized regression coefficient. Metal concentrations were log-transformed prior to regression analyses. *p < 0.05

**Table 8:** Determinants of Pb, Mn and Hg levels in BM above reference limits based on X² and ULR

	**Pb > LMT (5 µg/L)**	**Pb < LMT (5 µg/L)**	***p*** **-value**	**OR (95% CI)**
	***n*** ** (%)**	***n*** ** (%)**		
**Waste treatment**			0.047	
Collected by public services	34 (89.5)	4 (10.5)		Ref. 5.7 (1.1–29.1)
Waste burning	6 (60.0)	4 (40.0)		
**House Water Supply**			0.006	
Public water system	35 (89.7)	4 (10.3)		Ref.
Artesian wells and rivers	2 (33.3)	4 (66.7)		0.06 (0.01–0.42)
	**Mn > LMT (4.0 µg/L)**	**Mn < LMT (4.0 µg/L)**		
	**n (%)**	**n (%)**		
**Family income**			0.046	
> 1 salary	14 (87.5)	2 (12.5)		Ref.
≤ 1 salary	16 (55.2)	13 (44.8)		5.7 (1.1–29.7)
**Milk and dairy products intake**			0.029	
> 1x/week	23 (71.9)	9 (28.1)		**
< 1x/week		2 (100)		
	**Hg > LMT (1.7 µg/L)**	**Hg < LMT (1.7 µg/L)**		
	**n (%)**	**n (%)**		
**Municipality**			0.019	
Nazaré	16 (61.5)	10 (38.5)		Ref.
Aratuípe	20 (90.9)	2 (9.1)		6.3 (1.2–32.7)

**Odds ratio could not be calculated due to a cell count < 1

For Pb concentrations, household water supply was significantly associated with breast milk Pb levels after multivariate adjustment. Women whose households were supplied by the public water supply system had higher Pb concentrations than those using artesian well water (β = 1.048; 95% CI: 0.105–1.992). Household waste treatment also demonstrated a borderline association with Pb concentrations (β = 0.852; 95% CI: -0.055–1.759; *p* = 0.065). Complementary logistic regression analyses presented similar patterns of association for Pb concentrations above the international reference limit [27]. 

Regarding manganese (Mn) concentrations, both household waste treatment and domestic water supply remained significantly associated with Mn concentrations in breast milk in multivariate linear regression models. Women who reported household waste burning presented higher Mn concentrations in breast milk (β = 1.338; 95% CI: 0.697–1.979; *p* = 0.001), while the use of water from the public supply system was also associated with increased Mn concentrations (β = 0.943; 95% CI: 0.194–1.691; *p* = 0.015). Family income and consumption of milk and dairy products were not significantly associated with Mn concentrations after multivariate adjustment. However, in complementary logistic regression analyses, family income and consumption of milk and dairy products were more likely to be associated with Mn concentrations above the proposed reference limit.

For Hg concentrations, municipality of residence was significantly associated with BM Hg levels after multivariate adjustment. Women living in Aratuípe had higher Hg concentrations than those living in Nazaré (β = 0.824; 95% CI: 0.157–1.491; *p* = 0.017). In addition, complementary logistic regression analyses showed that lactating women living in Aratuípe were 6.3 times more likely (95% CI: 1.2–32.7) to have elevated Hg levels in their breast milk than those living in Nazaré.

## Discussion

The present study identified a pattern of multiple exposure to PTMs in breast milk and showed that selected environmental and contextual characteristics were associated with Pb, Mn, and Hg concentrations. The high frequency of concentrations above the adopted reference values, together with the observed correlations among some metals, indicates that maternal exposure in this population is better understood as a co-exposure scenario rather than as isolated exposure to individual contaminants. These findings provide the basis for the following discussion of metal concentrations, potential exposure pathways, and their toxicological implications.

### Levels of Metals in Breast Milk: Comparison and Toxicological Interpretation

The concentration profile of potentially toxic metals (PTMs) observed in the present study reveals a scenario of co-exposure to multiple PTMs among lactating women from the DSAN-12 M birth cohort. Lead (Pb) and mercury (Hg) presented the highest median concentrations, both exceeding internationally proposed reference values, while manganese (Mn) concentrations also surpassed the suggested threshold in a substantial proportion of samples. Although cadmium (Cd) concentrations were comparatively lower, approximately one quarter of the participants still presented levels above the recommended limit, indicating that exposure to multiple metals rather than to isolated contaminants may represent the predominant exposure pattern in this socially vulnerable population. Similar patterns of co-exposure have been reported in environmentally vulnerable populations worldwide, reinforcing that breast milk reflects the combined influence of maternal body burden and local environmental conditions rather than a single exposure source [4, 8, 11]. The significant correlations observed between metals further support the possibility of multiple exposure pathways. However, correlations between individual metals do not by themselves characterize the combined toxicological effect of the mixture.

The presence of PTMs in breast milk is biologically plausible because lactation represents a period of intense physiological adaptation, during which maternal mineral metabolism is substantially altered. Physiological bone resorption associated with the increased calcium demand of lactation promotes the mobilization of Pb stored in the maternal skeleton [4, 32–34], . Mercury can reach breast milk from the maternal blood circulation. Recent evidence indicates that maternal dietary exposure, particularly through seafood consumption, is associated with Hg concentrations in human milk, supporting the role of breast milk as a relevant postnatal exposure pathway [35, 36]. Arsenic is also detectable in human milk, although its transfer appears to be relatively limited and dependent on maternal exposure and chemical speciation. Recent studies indicate that maternal diet and environmental sources contribute to As concentrations in breast milk, although the secretion mechanisms into the mammary compartment remain poorly established [35, 36]. Experimental evidence further suggests that mammary epithelial cells express metal transport systems, including divalent metal transporter-1 (DMT1) and transferrin receptors, which may facilitate the transfer of essential trace elements into BM. Because toxic divalent metals such as Pb and Cd are able to utilize these transport pathways, they can be transferred into BM under specific physiological conditions [32, 33].

Consequently, breast milk constitutes an important biomonitoring matrix because it integrates both recent environmental exposure and particularly for Pb, the mobilization of maternally stored metals throughout life during lactation [4, 32]. Therefore, BM concentrations should be interpreted not only as indicators of infant exposure but also as biomarkers of cumulative maternal exposure and environmental contamination within the population under study [4, 5].

Comparison with previous Brazilian and international studies places the DSAN-12 M findings in the upper range of PTM concentrations reported in Brazil, yet remains lower than values reported in some environmentally contaminated regions worldwide. For example, Naspolini et al. reported a mean concentration of 2.76 µg/L for As, 2.09 µg/L for Pb, and 1.96 µg/L for Hg in BM samples of lactating women from the metropolitan region of São Paulo, whereas studies conducted in Minas Gerais reported considerably lower Pb concentrations (0.26 µg/L) [14, 37, 38]. In comparison, the DSAN-12 M cohort showed lower median As concentrations but considerably higher Pb and Hg concentrations, indicating a distinct regional exposure profile. Conversely, Pb concentrations approaching those observed in the present study have been reported in Brazilian human milk banks, and substantially higher levels have been described in populations exposed to intense environmental contamination, such as those reported in Nigeria [37–41]. These comparisons indicate that exceeding the proposed reference values is not unique to the DSAN-12 M population. However, the magnitude and combination of metals observed in the present study suggest a comparatively higher level of environmental exposure. These differences likely reflect the heterogeneous environmental conditions, exposure sources, analytical methodologies, and socioeconomic characteristics across study populations, highlighting the importance of interpreting breast milk metal concentrations within their specific environmental context rather than relying solely on direct numerical comparisons [8, 11].

Arsenic needs particular concern because, although it did not present the highest absolute median concentration among the metals evaluated, a large proportion of samples exceeded the reference value. Human milk: concentrations vary substantially among populations and may reflect both dietary and environmental exposures [35, 36]. Previous evidence has identified maternal food consumption and water source as relevant determinants of As exposure, while higher concentrations have also been reported in populations living in environmentally contaminated areas [35, 37]. The frequent occurrence of As concentrations above the adopted reference value in the DSAN-12 M cohort, therefore, suggests that specific environmental and dietary sources of As exposure should be further investigated in this population.

The simultaneous detection of multiple PTMs and the correlations observed among some metals suggest that maternal exposure in this population may involve multiple exposure pathways. Such a co-exposure pattern is particularly relevant in socially vulnerable settings, where different environmental sources may coexist. However, the present metal-specific analyses cannot determine the joint toxicological effects of these exposures. Future studies using analytical approaches specifically designed for environmental mixtures may therefore complement conventional single-metal evaluation.

### Prevalence of PTMs Concentrations Above the Reference Limits

A substantial proportion of lactating women enrolled in the DSAN-12 M birth cohort presented breast milk concentrations exceeding internationally proposed reference values for PTMs [31], particularly for As, Hg, Pb, and Mn. Overall, the prevalence observed in the present study was higher than that reported in most Brazilian and international investigations, suggesting that environmental exposure to multiple PTMs may be widespread in this socially vulnerable population rather than being restricted to a limited number of highly exposed individuals [8, 10, 39, 41, 42]. Nevertheless, the simultaneous occurrence of concentrations above reference values should not be interpreted as a quantitative estimate of cumulative toxicological risk, because the reference values used for individual metals exposure were not designed as toxicity benchmarks for constructing a multiple mixture-based risk index.

The predominance of As, Hg, and Pb concentrations above the proposed reference limits is consistent with reports from environmentally vulnerable regions where different environmental contamination sources, including artisanal lead-glazed ceramic production, contaminated drinking water, industrial emissions, mining activities, and diffuse environmental pollution contribute to chronic human exposure [13, 26, 42]. Likewise, the high proportion of Mn concentrations exceeding the suggested threshold warrants attention, as Mn, although essential for normal growth and neurodevelopment, may exert neurotoxic effects when exposure exceeds physiological requirements [43, 44]. Rather than representing isolated contamination events, the simultaneous occurrence of elevated concentrations of multiple PTMs supports the hypothesis that maternal exposure in the studied population arises from multiple environmental pathways.

### Implications of BM Contamination for Infant Health and Development

The exposure profile observed in the present study raises concern because breastfed infants may be simultaneously exposed to multiple PTMs during a critical period of rapid brain growth and neurodevelopment. Although breastfeeding remains the optimal source of infant nutrition and its benefits unquestionably outweigh the potential risks associated with environmental contaminants, elevated concentrations of Pb, Hg, and Mn warrant particular attention because these elements can cross the immature blood–brain barrier and interfere with key neurodevelopmental processes [37, 45]. Pb and Hg are well-established developmental neurotoxicants, whereas excessive Mn exposure, despite its essential physiological role, has also been associated with impaired motor development, cognitive dysfunction, and behavioral alterations in children [46–48].

In addition to their individual toxicological effects, increasing evidence suggests that co-exposure to multiple PTMs may produce biological responses that cannot be explained by single-metal toxicity alone. Experimental and epidemiological evidence indicates that co-exposure to multiple PTMs may promote oxidative stress, chronic inflammation, and other biological disturbances that contribute to adverse health outcomes. These effects are mediated through multiple interacting toxicological pathways, including alterations in cellular redox homeostasis and inflammatory signaling [13, 47–50]. Importantly, studies of early-life and adolescent exposure to metal mixtures have shown that neurodevelopmental associations may differ from those observed when metals are evaluated individually, supporting the need to consider mixture effects when investigating neurodevelopmental outcomes [51, 52]. Future investigations integrating breast milk metal concentrations with prospective neurodevelopmental assessments and multiple mixture-based statistical approaches may provide a more comprehensive understanding of the health implications of early-life co-exposure.

### Pb, Mn, and Hg in BM and its Determinants

Unlike many previous studies that have primarily focused on maternal dietary habits or individual socioeconomic characteristics as determinants of potentially toxic metal (PTM) concentrations in breast milk [10, 14, 37, 41, 42], the present study identified structural environmental conditions as the principal determinants of maternal exposure to Pb, Mn, and Hg. Household water supply, household waste burning, and municipality of residence remained independently associated with breast milk metal concentrations after multivariable adjustment, suggesting that environmental infrastructure and local environmental characteristics may exert a greater influence on maternal body burden than individual behavioral factors in this socially vulnerable population. While several studies have described PTM concentrations in human milk, comparatively fewer investigations have explored the environmental determinants underlying maternal-to-infant transfer during lactation, particularly in prospective birth cohorts from low- and middle-income countries [5, 7, 8].

Among the identified determinants, household water supply emerged as an independent predictor of Pb concentrations in breast milk. Women whose households relied on the public water supply system presented significantly higher Pb concentrations than those using artesian wells, suggesting that drinking water or characteristics of the distribution network may represent an important environmental exposure pathway in the study area. Although treated public water generally complies with drinking water quality standards, and is generally considered safe, Pb contamination may occur ater treatment, during distribution or within household plumbing, particularly through corrosion and leaching from Pb-containing plumbing materials, solder joints, fittings, or legacy components of water distribution systems [53–56]. Although treated drinking water may become contaminated through corrosion within distribution systems [53, 54, 56], studies from environmentally vulnerable low- and middle-income settings indicate that drinking water may represent one of several environmental pathways contributing to human Pb exposure [8, 11]. These mechanisms may be particularly relevant in settings with aging or heterogeneous water infrastructure. However, as Pb concentrations in drinking water and characteristics of the local distribution network were not directly assessed in the present study, the observed association should not be interpreted as direct evidence of contamination of the municipal water supply. Rather, it identifies public water supply as a potential marker of an environmental exposure pathway that warrants further investigation.

The observed association is biologically plausible because chronic ingestion of Pb-contaminated water contributes to cumulative maternal body burden. During pregnancy and lactation, physiological bone resorption associated with increased calcium demand mobilizes previously stored Pb from maternal bone, facilitating its transfer into breast milk even when current environmental exposure is relatively low [32].

Household waste burning also emerged as an important environmental determinant of breast milk metal concentrations, particularly for Mn and, to a lesser extent, Pb. Although the association with Pb was only of borderline statistical significance after multivariable adjustment, the consistent association observed for Mn strengthens the hypothesis that open waste burning represents a relevant source of environmental exposure in the study area. Burning household waste is recognized as an important source of airborne particulate matter enriched with trace elements and other toxic pollutants, which may subsequently contaminate soil, household dust, surface water, and locally produced foods through atmospheric deposition [57]. Consequently, human exposure may occur simultaneously through multiple pathways, including inhalation of contaminated particles, incidental ingestion of contaminated dust, and dietary intake of contaminated food and water [4, 57, 58].

This exposure scenario is particularly plausible in socially vulnerable communities, where deficiencies in public waste collection services often increase the routine practice of waste burning. Repeated low-level environmental exposure resulting from this practice may progressively increase maternal body burden over time, thereby contributing to the accumulation of metals that can subsequently be mobilized during pregnancy and lactation and transferred into breast milk [32]. Similar associations between informal waste burning, environmental contamination, and increased human exposure to trace elements have been reported in Brazil, Bangladesh, and China, supporting the biological plausibility of the associations observed in the present study [45, 59, 60].

The findings for Mn further reinforce the predominant role of environmental determinants in shaping maternal exposure within the study population. Unlike Pb, for which the association with household waste burning showed only borderline significance after multivariable adjustment, both household waste burning and reliance on the public water supply remained independently associated with Mn concentrations in breast milk. These results suggest that Mn exposure may be more consistently influenced by chronic environmental sources than by individual behavioral factors, supporting the hypothesis that environmental infrastructure contributes substantially to maternal Mn exposure [37, 61].

An additional finding deserving attention was the loss of significance of family income and dairy product consumption after multivariable adjustment. Although these variables presented associations in the univariate analyses, their effects were no longer observed after adjustment for environmental factors, suggesting that the initial associations were likely influenced by confounding. Similar studies have shown that PTM concentrations in human milk may reflect the combined influence of maternal dietary and socioeconomic characteristics, as well higher environmental exposure conditions [10, 37, 41]. In environmentally vulnerable populations, characteristics of the environment, including contamination of water, soil, and other local exposure sources, have also been associated with maternal metal exposure [37, 42, 62].This finding highlights the importance of considering structural environmental characteristics when investigating maternal exposure to trace elements, as environmental determinants may outweigh the influence of dietary or socioeconomic variables in socially vulnerable settings.

Because Mn is an essential trace element required for normal growth and neurodevelopment, public health interventions should not focus on reducing Mn exposure indiscriminately, but rather on identifying and mitigate excessive environmental exposure while preserving adequate nutritional intake. This distinction is particularly important during pregnancy and lactation, when both Mn deficiency and excessive exposure may adversely affect infant development [63, 64].

For Hg, municipality of residence emerged as the only independent determinant of breast milk concentrations, with women living in Aratuípe presenting significantly higher Hg levels than those residing in Nazaré. Interestingly, seafood consumption did not differ significantly between municipalities, suggesting that dietary habits alone are unlikely to explain the observed differences in breast milk Hg concentrations. Environmental processes such as Hg mobilization from sediments, interactions with organic matter, hydrological conditions, atmospheric deposition, waste disposal, domestic effluents, or other diffuse sources may influence Hg environmental exposure. Although the present study was not designed to identify specific environmental Hg sources, the observed statically significant difference between municipalities highlights the importance of region-specific environmental surveillance and targeted biomonitoring strategies. Future investigations integrating environmental sampling, geospatial analyses, and human biomonitoring may help identify local Hg sources and clarify the environmental pathways contributing to maternal exposure in low-income communities.

The consistent associations observed for household water supply, household waste burning, and municipality of residence suggest that environmental infrastructure and local environmental context play a central role in chronic maternal exposure and, consequently, infant exposure through breast milk.

### Strengths, Limitations, and Health Perspectives

As a prospective cohort study, this work presents important strengths, including the temporal separation between exposure assessment during pregnancy and breast milk collection after delivery. This design strengthens the interpretation of associations between sociodemographic, environmental, and dietary factors and metal concentrations in breast milk. Another important strength is the focus on women who maintained exclusive breastfeeding at the time of sample collection, which provides a more specific estimate of infant exposure to PTMs through breast milk during an important developmental period. The study also provides a systematic assessment of potentially toxic metals in breast milk from women living in the Recôncavo Baiano region, a socially vulnerable and under-monitored area of Brazil. The application of the ICP-MS/MS method ensured high analytical sensitivity and accuracy, while integrating sociodemographic, dietary, and environmental variables enabled investigation of multiple potential determinants of maternal exposure to PTMs.

However, some limitations should be considered. Breast milk samples were collected only from women who were maintaining exclusive breastfeeding at the time of collection. Therefore, the reduced number of samples analyzed does not reflect random loss to follow-up but rather the application of biological and methodological criteria. The relatively small number of breast milk samples may have reduced the statistical power of the analyses and the precision of some estimates. In addition, environmental concentrations of PTMs in water, soil, sediment, air, or food were not measured directly, limiting the ability to identify specific contamination sources or to establish causal exposure pathways.

From a public health perspective, the findings highlight the need to strengthen human biomonitoring and environmental surveillance among maternal and child populations living in socially vulnerable conditions. Future studies integrating human biomonitoring with direct environmental measurements and child health and neurodevelopmental outcomes will be important to clarify exposure pathways and the potential consequences of early-life co-exposure to PTMs.

## Conclusions

This study identified widespread exposure to potentially toxic metals among lactating women enrolled in the DSAN-12 M birth cohort in the Recôncavo Baiano region of Brazil. The levels of As, Hg, Mn and Pb in breast milk frequently exceeded internationally proposed reference values, indicating simultaneous exposure to multiple PTMs rather than isolated exposure to individual metals in a population characterized by socioeconomic vulnerability.

The observed exposure pattern suggests that structural and contextual environmental conditions play an important role in maternal exposure during lactation. Particularly, characteristics related to household water supply, waste management practices, and place of residence may reflect environmental pathways contributing to differences in breast milk metal concentrations. However, because environmental concentrations and specific contamination sources were not directly measured, these associations should be interpreted cautiously and should not be considered evidence of causal exposure pathways.

Although breastfeeding remains the optimal and strongly recommended practice for infant feeding, these findings should not be interpreted as discouraging breastfeeding but rather highlighting the importance of reducing maternal exposure to PTMs through environmental and public health policies and interventions.

Overall, the findings indicate that maternal exposure to PTMs in this socially vulnerable population is related to the environmental context in which women live, emphasizing the importance of strengthening environmental surveillance, water-distribution infrastructure, waste-management practices, and human biomonitoring. Future longitudinal investigations integrating human biomonitoring, environmental, mixture-based approaches and child neurodevelopment outcomes are necessary to further elucidate exposure pathways and the long-term health implications of early-life co-exposure to potentially toxic metals.

## Data Availability

The data generated and analyzed during this study are not publicly available because they contain confidential information from research participants and are protected by ethical restrictions. However, the data may be made available by the corresponding author upon reasonable request and with permission from the Research Ethics Committee.
